# Retisert implantation without incisional sclerotomy in patients with uveitis and extensive pars plana fibrosis

**DOI:** 10.1016/j.ajoc.2024.102135

**Published:** 2024-07-31

**Authors:** Abu Tahir Taha, Joshua Wu, Julie M. Schallhorn, Jay M. Stewart

**Affiliations:** a University of California, Department of Ophthalmology, San Francisco, CA, USA; b Zuckerberg San Francisco General Hospital and Trauma Center, Department of Ophthalmology, San Francisco, CA, USA; c University of California, Francis I. Proctor Foundation, San Francisco, CA, USA

**Keywords:** Retisert, Uveitis, Fibrosis

## Abstract

**Purpose:**

To describe an alternate surgical technique for fluocinolone acetonide (Retisert) implantation in patients with extensive pars plana and pars plicata fibrosis secondary to chronic non-infectious uveitis.

**Methods:**

This retrospective, interventional case series included five eyes of four patients who had poorly controlled chronic non-infectious uveitis. Retisert was implanted successfully using a novel approach. The device was introduced into the posterior segment through the anterior chamber and posterior capsulotomy, forgoing the need for full-thickness scleral incision and minimizing the risk of retinal detachment and associated complications.

**Results:**

Five eyes underwent passage of Retisert implant through the anterior segment via a limbal incision and a posterior capsulotomy. Retisert was successfully implanted in all patients in the posterior chamber. No intraoperative or postoperative complications were encountered. Up until the last follow-up, all eyes demonstrated the stability of the implant. Visual acuity improved in four out of five eyes.

**Conclusions:**

Retisert can be implanted via the anterior chamber in patients with extensive fibrosis in the pars plana and pars plicata regions. This approach may minimize the risk of retinal traction and damage to the implant when compared to the traditional full-thickness sclerotomy method in these high-risk cases.

## 1 Introduction

Chronic non-infectious uveitis (NIU) can affect people of any age and carries significant ocular morbidity.^1^ Uveitis of any anatomic location (anterior, intermediate, and posterior) can involve inflammation of several ocular structures, resulting in choroiditis, pars planitis, posterior cyclitis, and hyalitis.^2^^,^^3^ Chronic flares of inflammation in these areas can result in proliferative fibrosis,^4^ making future surgical interventions via the pars plana technically challenging. The goal of treatment in patients with NIU is to control inflammation, which is usually accomplished by employing a treatment ladder of topical, systemic, or intravitreal corticosteroids paired with immunosuppressive agents.^3^ Intraocular steroid implants are often used in the later part of the treatment regimen for patients who continue to fail or are intolerant of systemic and topical therapy.

One such implant is the fluocinolone acetonide intravitreal implant (Retisert), which can deliver steroids at a rate of 0.3–0.4 μg/day for approximately 2.5 years.^3^ Studies have shown that Retisert reduces posterior uveitis recurrence in adult and pediatric chronic NIU patients.^5^^,^^6^ The placement of a Retisert implant requires a transscleral incision of at least 3.0 mm in length, through which the implant is passed into the vitreous cavity.^7^ However, patients with dense fibrovascular membrane formation in the pars plana and pars plicata regions from longstanding inflammation may be at higher risk of adverse events such as choroidal or retinal detachment.^8^ Moreover, insertion of the implant through a fibrotic tissue may increase shearing forces on the implant, increasing the chances of perforation of the membrane covering the implant or separation of its components,^9^ which may affect the rate of drug delivery.^7^ This brief report describes an alternate approach to Retisert implantation that may minimize the risks of the aforementioned complications.

## 2 Material and methods

### 2.1 Surgical technique

**Fig. 1:**
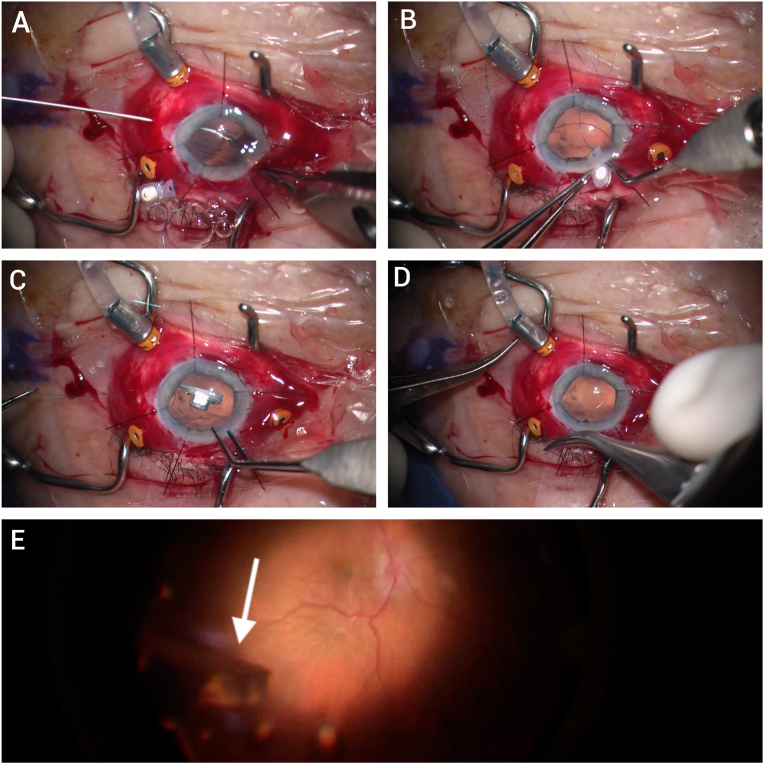
Title: A panel of video stills showing key steps of Retisert insertion from the anterior chamber.

Intraoperative endoscopy was used to confirm the presence of extensive pars plana fibrosis in all patients. In phakic eyes, the first step was the synechiolysis of any bridging membranes between the iris and the anterior chamber, followed by phacoemulsification to remove the lens, leaving the posterior capsule intact. Pars plana vitrectomy was performed, followed by a primary posterior capsulotomy. The main limbal incision was enlarged to 3.5 mm to accommodate the Retisert implant. The implant was prepared off the surgical field by tying an 8-0 Prolene suture through the hole in the plate. After creating a peritomy, a 27-gauge needle was inserted transsclerally 3.5 mm posterior to the limbus and then removed to create a needle-sized sclerotomy. Retinal 27-gauge forceps were then inserted through the sclerotomy into the vitreous cavity, grasping one of the sutures attached to the Retisert that was introduced into the anterior chamber through the corneal incision and then advanced posteriorly through the capsulotomy (Fig. 1). The suture was then externalized through the sclerotomy. The second suture attached to the Retisert was also externalized similarly through a separate sclerotomy that was made adjacent to the first one. The implant was inserted into the anterior chamber and through the posterior capsulotomy. The two sutures externalized through the sclerotomies were then pulled in order to deliver the implant through the posterior capsulotomy into the posterior chamber and to its final position against the pars plana. Key steps of this procedure can be observed in Fig. 1 and the attached video (Video 1).

Supplementary video related to this article can be found at https://doi.org/10.1016/j.ajoc.2024.102135

The following is/are the supplementary data related to this article:

Multimedia component 2 Multimedia component 2

## 3 Video captions

Video 1: Anterior segment approach to Retisert implantation. Phacoemulsification as well as lysis of membranes in the anterior chamber is performed. An endoscope is used to visualize the pars plana regions showing diffuse fibrosis. A primary posterior capsulotomy is made. Two sclerotomies in the limited temporal peritomy region are made, and both sutures attached to the Retisert are externalized through the sclerotomies. The Retisert implant is then inserted into the anterior chamber through the clear corneal incision. The externalized sutures are pulled back, taking the implant from the anterior chamber through the posterior capsulotomy into the posterior chamber. The implant is then secured in the vitreous cavity.

## 4 Results

This technique for Retisert implantation has been used in five eyes of four patients. It was combined with additional procedures such as silicone oil injection to address hypotony. Four out of five eyes were phakic with visually significant cataracts and underwent cataract removal with primary posterior capsulotomy to facilitate Retisert implantation. All patients recovered well without any intraoperative or post-operative complications. A more comprehensive clinical history of each patient and their medical management before Retisert implantation can be viewed in Supplemental Table S1. Visual acuity, intraocular pressure (IOP), and longest follow-up interval for each patient before and after the surgery are listed in Table 1. All patients, except patient 4, experienced improvement in their vision. All patients displayed quiescence of uveitis at their most recent follow-up visit.

**Table 1:** Post-operative outcomes after implantation of Retisert without incisional sclerotomy.

Patient age and sex	Procedure performed	Preop uveitis status	Preop corrected vision	Preop IOP (mmHg)	POW1 vision	POW1 IOP (mmHg)	POM1 vision	POM1 IOP (mmHg)	Most recent vision	Most recent IOP (mmHg)	Longest follow up time	Most recent uveitis status	Notes/Postoperative complications
77, Female	PPV/Retisert/IOL in sulcus with capsular tension ring	Quiet	CF 1ft	2	CF 4ft	3	20/125	5	20/150	3	7 months	Quiet	Patient underwent SO injection POM5 for persistent hypotony
45, Female	PPV/Retisert/SO/IOL in capsular bag	Unable to assess^a^	CF 1ft	2	20/100	6	20/150	6	20/125	9	8 months	Quiet	
16, Male OD	PPV/Retisert/EL/SO/IOL in sulcus with optic capture	1+ flare	HM	3	–	–	20/100	3	20/150	3	6 months	Quiet	Patient planned for a removal of membrane across IOL in the OR
16, Male OS	PPV/Retisert/EL/SO/IOL in sulcus	1+ flare; trace pigment	HM	3	–	–	20/200	5	20/150	3	3 months	Quiet	Fibrotic membrane between IOL and iris; visually insignificant
13, Female	PPV/Retisert/EL/Retinectomy/SO	Flare; no cell	CF 3ft	2	CF 1ft	8	CF 1ft	Soft	CF 1ft	6	6 months	Quiet	

a Patient presented with iris apposed to the peripheral cornea, making it difficult to assess the level of inflammation. Preop = pre-operative, IOP = intraocular pressure, IOL = intraocular lens, PPV = pars plana vitrectomy, SO = silicone oil, OR = operating room, EL = endolaser, HM = hand motion, CF = counting fingers, POW1 = postoperative 1 week, POM1 = postoperative 1 month, - = not available.

## 5 Discussion

This alternate approach to Retisert implantation can be utilized in cases in which extensive fibrovascular proliferation in the pars plana and pars plicata regions is present. This diagnosis is usually formed with ultrasound biomicroscopy (UBM)^10^^,^^11^; therefore, intraoperative endoscopy is discretionary and not a prerequisite to determine whether this approach would benefit a patient or to perform this procedure safely. An essential part of our technique is avoiding a full-thickness transscleral incision, which may prevent tugging on the retina and reduce the risk of rhegmatogenous or tractional retinal detachment in this scenario.^12^ Patients with prior extensive ocular surgeries and scleral thinning may also benefit from this technique, as it depends less on the integrity of overlying Tenon's capsule and conjunctiva since there is not the usual full-thickness scleral incision 3–4 mm in length.^13^

The most common adverse effects of Retisert implantation are cataract progression and increased IOP.^14^ However, our patients presented with hypotony, a known complication of chronic intraocular inflammation.^15^ This was likely due to chronic iridocyclitis, detachment of the ciliary body, or formation of cyclitic membranes, as documented in Supplemental Table S1. The persistence of hypotony in 4 out of 5 eyes despite silicone oil instillation may be attributed to the fact that it can take six months or longer for eyes to experience substantial increases in IOP.^16^ Therefore, it is possible that a longer follow-up may reveal elevation of IOP in our cases – which would be a desired outcome in our study. All operated eyes, barring one, experienced an improvement in their visual acuity (VA) – from hand motion (HM) and counting fingers (CF) to 20/125, likely as a result of the combination of procedures performed, including Retisert implantation and silicone oil injection, with the latter potentially helping structural hypotony.^15^ Patient 4 (13 y.o, female) had extensive tractional retinal detachment that was noted over one month in advance of the procedure, which may have limited the visual gain after the surgery. A year before her Retisert surgery, she underwent cataract extraction and was left aphakic. At the time of Retisert implantation, she had unresolved band keratopathy and uncertain health of the retina, thus, a decision was made to forgo intraocular lens insertion and use a scleral contact lens in the near future. Case 3 (16 y.o.) re-developed membranes across the IOL (OD) and between the iris and anterior capsule (OS) after the surgery, a phenomenon that has previously been reported in up to 44 % of eyes of a pediatric cohort with juvenile idiopathic arthritis (JIA) associated uveitis who underwent cataract surgery. Overall, our technique displayed long-term stability and functionality of the Retisert implant as evidenced by patients’ quiet uveitis status and improved vision at their most recent follow-up visits. If necessary in the future, we intend to exchange the Retisert implant by first removing the IOL and then removing the implant through the posterior capsulotomy. The new Retisert would be implanted using the same technique mentioned in this report, and the IOL would be re-inserted subsequently.

## 6 Conclusions

Patients with extensive fibrosis of pars plana and pars plicata regions from chronic NIU may benefit from an alternative technique of Retisert insertion. This technique inserts the implant into the anterior chamber and through the posterior capsulotomy to minimize the risk of retinal complications and failure of the implant.

## Patient consent

Informed consent was not obtained from the patients to publish this retrospective report. All patients provided full consent for surgery. This report does not contain any personal information that could lead to the identification of the patient.

## Research ethics

The University of California, San Francisco Institutional Review Board reviewed and approved this case series.

## Funding

This report was supported by Research to Prevent Blindness Unrestricted Grant to the UCSF Department of Ophthalmology, and All May See Foundation, San Francisco, CA.

## CRediT authorship contribution statement

**Abu Tahir Taha:** Writing – original draft, Investigation. **Joshua Wu:** Writing – review & editing, Resources, Data curation. **Julie M. Schallhorn:** Writing – review & editing, Resources, Methodology. **Jay M. Stewart:** Writing – review & editing, Resources, Methodology, Investigation, Funding acquisition, Conceptualization.

## Declaration of competing interest

The authors declare that they have no known competing financial interests or personal relationships that could have appeared to influence the work reported in this paper.
