# Supplementary material for: Retisert implantation without incisional sclerotomy in patients with uveitis and extensive pars plana fibrosis
Source: Am J Ophthalmol Case Rep. 2024 Jul 31;36:102135. doi: 10.1016/j.ajoc.2024.102135 (PMC11347055; doi:10.1016/j.ajoc.2024.102135)
Supplement: Multimedia component 1 [file mmc1.docx]

| **Table S1. Clinical history and pre-surgical management details of patients who underwent Retisert implantation** | | | | |
| --- | --- | --- | --- | --- |
| **Patient information and history of uveitis** | **Medical therapies and interventions prior to Retisert implantation** | **Preoperative intraocular pressure (mmHg)** | **Uveitis related ocular morbidities present before surgery** | **Intra and post-operative complications and additional interventions** |
| 77-year-old female with checkpoint inhibitor associated bilateral anterior-intermediate uveitis present for 11 months prior to surgery. Patient’s uveitis was quiescent at least 4 months prior to surgery. | Patient was using 50 mg oral steroids for pneumonitis and difluprednate drops for uveitis. Patient previously received one-time sub-Tenon triamcinolone acetonide injections bilaterally. Despite this regimen, the hypotony persisted bilaterally. | 2 | Bilateral hypotony, hypotony maculopathy, cataract, dense cyclitic membranes that were contiguous with the lens capsule. | No intra-operative complications were encountered. Patient received additional silicone oil 5 months post-operatively for continued hypotony. |
| 45-year-old female with bilateral undifferentiated necrotizing sclerokeratitis and iridocyclitis starting at age 35, controlled with immunotherapy at the time of surgery. Five months prior to surgery, she developed a scleral perforation that was patched with persistent hypotony. At time of surgery, her anterior chamber was completely collapsed. | Patient intermittently used oral steroids during scleromalacia flares in the past 10 years with along with continuous usage of immunotherapy. At the time of pre-operative evaluation, she was using prednisolone acetate drops 6 times per day in the affected eye. | 2 | Epiciliary membranes visible on ultrasound biomicroscopy. Posterior synechiae. Vitreous membranes. Persistent hypotony. Dense cataract. | None |
| 16-year-old male with juvenile idiopathic arthritis and uveitis starting at age 5 with persistent hypotony since 2019. | Previously used methotrexate, infliximab, oral steroids, and prednisolone acetate drops. Quiescence was achieved in 2014 to 2015 on methotrexate and infliximab. In 2021 patient switched to tocilizumab. | OD: 3, OS: 3 | OD: Pupillary membranes. Vitreous membranes. Resorbed lens. Anterior retina tractional detachment. Persistent hypotony.  OS: Pupillary membranes. Dense fibrotic cataract. Inferior tractional retinal folds. Persistent hypotony. | Re-formation of dense membranes across IOL OD and fibrotic membrane between iris and IOL OS – the former may need membranectomy; the latter is visually insignificant. |
| 13-year-old female was diagnosed with chronic undifferentiated bilateral iridocyclitis starting at age 4. After achieving quiescence in 2022, she underwent OD phacoemulsification and was left aphakic, with postoperative hypotony and choroidal detachments. She subsequently developed a tractional retinal detachment that required surgical repair. | The inflammation was refractory to Infliximab and adalimumab. She achieved quiescence on methotrexate, tocilizumab, and prednisone. | 2 | Posterior synechiae and inferior retinal detachment along with choroidal folds. Ciliary body detachment. | none |
